# The Impact of Induction With Azacitidine and Venetoclax Versus Intensive Chemotherapy on Outcomes After Allogeneic Hematopoietic Cell Transplantation for Newly Diagnosed Acute Myeloid Leukemia

**DOI:** 10.14740/jh2217

**Published:** 2026-08-31

**Authors:** Baldeep Wirk, Rami Hawila

**Affiliations:** a Department of Cellular Immunotherapies and Transplant, Massey Comprehensive Cancer Center, Virginia Commonwealth University, Richmond, VA, USA; b Department of Biostatistics, School of Public Health, Virginia Commonwealth University, Richmond, VA, USA

**Keywords:** Acute myeloid leukemia, Azacitidine, Venetoclax, Intensive chemotherapy, Allogeneic hematopoietic cell transplantation

## Abstract

**Background:**

Induction with azacitidine and venetoclax (AZA-VEN) is standard therapy for patients 75 years or older or those with comorbidities that preclude intensive chemotherapy (IC) for acute myeloid leukemia (AML). Data on the impact of AZA-VEN on allogeneic hematopoietic cell transplant (HCT) outcomes in younger, fit patients are limited.

**Methods:**

In a single-center retrospective study, we examined adult patients with newly diagnosed AML receiving either AZA-VEN or IC (cytarabine and anthracycline) to attain first complete remission (CR) before allogeneic HCT with posttransplant cyclophosphamide-based graft-versus-host disease (GVHD) prophylaxis between 2023 and 2025.

**Results:**

The median age in the AZA-VEN (N = 14) and IC (N = 17) groups was similar (67 years (62–69) vs. 63 years (54–66), P = 0.13). The AZA-VEN group had more adverse-risk AML (93% vs. 41%, P = 0.001). Otherwise, there were no significant differences in Hematopoietic Cell Transplantation Comorbidity Index, secondary AML incidence, minimal residual disease (MRD) status, or donor types between the two cohorts. The AZA-VEN and IC cohorts had similar 1-year overall survival (69.2%, 95% confidence interval (CI) (48.2–99.5) vs. 75%, 95% CI (56.5–99.7), P = 0.73), 1-year relapse-free survival (57.1%, 95% CI (36.3–89.9) vs. 64.7%, 95% CI (45.5–91.9), P = 0.67), 1-year GVHD-free relapse-free survival (57.1%, 95% CI (36.3–89.9) vs. 64.7% 95% CI (45.5–91.9), P = 0.67), 1-year cumulative incidence of relapse (14.3% vs. 11.8%, P = 0.84), 1-year cumulative incidence of non-relapse mortality (28.5% vs. 23.5%, P = 0.76), 1-year cumulative incidence of grade 3–4 acute GVHD (14.3% vs. 5.9%, P = 0.46), and 1-year cumulative incidence of chronic GVHD (7.1% vs. 11.8 %, P = 0.67). On multivariable Cox analysis, there was no significant impact of treatment cohort (AZA-VEN vs. IC), MRD, adverse-risk genetics, acute GVHD, Hematopoietic Cell Transplantation Comorbidity Index, or age on overall survival.

**Conclusions:**

In newly diagnosed AML, AZA-VEN and IC induction yield comparable outcomes after allogeneic HCT, regardless of age or adverse genetics, warranting prospective studies.

## Introduction

Since 1975, the 7 + 3 regimen—cytarabine with an anthracycline—has been the standard first-line therapy for newly diagnosed acute myeloid leukemia (AML). Cytarabine with either daunorubicin or idarubicin offers a 40% cure rate in patients younger than 60 years fit enough to receive intensive chemotherapy (IC), consolidation, and allogeneic hematopoietic cell transplantation (HCT) [1]. Patients over 60 or unfit for IC have a median survival of 9 months and a 5-year survival of 15% [2]. Notably, 68 years is the median age of onset of AML [3]. Older patients are not only more likely to be ineligible for IC but also have adverse-risk genetics with a higher incidence of secondary AML [3].

Pivotal studies of induction chemotherapy excluded older patients, those with comorbidities, or patients with secondary AML evolving from myelodysplastic syndrome (which accounts for 20% of AML) treated with hypomethylating agents [1]. Notably, patients over 70 or medically unfit for IC represent 30–40% of AML cases. For these older or unfit AML patients, the median overall survival (OS) with supportive care was 3 months, before the introduction of azacitidine and venetoclax (AZA-VEN) [4].

AML blasts exhibit increased expression of B-cell lymphoma 2 (BCL2) proteins, which promote cell survival by inhibiting mitochondrial apoptotic signaling [5]. Increased BCL2 expression promotes chemoresistance in AML [6]. Venetoclax, a selective small-molecule BCL2 inhibitor, induces apoptosis in AML blasts that depend on BCL2 for survival [7]. Although venetoclax alone has limited activity in AML, azacitidine synergistically inhibits the anti-apoptotic proteins myeloid cell leukemia 1 (MCL1) and BCL-XL. This dual inhibition forces leukemia cells to rely predominantly on BCL2 for survival, making them more susceptible to BCL2 blockade by venetoclax [7].

The VIALE-A study showed that AZA-VEN had a 14.7-month median OS compared with 9.6 months with azacitidine in older/unfit patients with newly diagnosed AML [8]. Azacitidine-venetoclax was granted accelerated approval in November 2018, with full approval in October 2020, as induction therapy for newly diagnosed AML patients 75 years or older or those unfit for IC due to comorbidities.

AZA-VEN induction enables older patients with AML to have a potentially curative allogeneic HCT. For instance, in AML patients over 60, 20% underwent transplant following AZA-VEN induction, and survival was improved in transplanted patients compared with non-transplanted eligible patients (median not reached vs. 518 days, P = 0.01) [9]. Building on these findings, PARADIGM, a phase 2 randomized multicenter study, compared AZA-VEN with IC in newly diagnosed fit AML patients, regardless of age. The doublet AZA-VEN achieved a greater composite remission rate (81% vs. 55%, P < 0.001) and lower 60-day mortality (0% vs. 4.7%) than IC [10]. Further, the 1-year event-free survival was better in the AZA-VEN group than in the IC group (53% vs. 39%, P = 0.017). The AZA-VEN group had fewer inpatient days (15 vs. 86, P < 0.001), fewer intensive care transfers (0 vs. 9.8%, P = 0.003), and improved quality of life (P = 0.001). As a result, more patients in the AZA-VEN arm underwent transplantation than in the IC arm (60% vs. 40%, P = 0.009) [10].

Data on the impact of induction therapy with either AZA-VEN or IC on allogeneic HCT outcomes are scarce, with conflicting results [11–13]. We examined the outcomes in patients aged 18 or older with AML who received either AZA-VEN or IC (cytarabine and anthracycline) to achieve first complete remission (CR) before undergoing allogeneic HCT. All patients received posttransplant cyclophosphamide, mycophenolate mofetil, and a calcineurin inhibitor for graft-versus-host disease (GVHD) prophylaxis.

## Materials and Methods

### Patients

We reviewed patients aged 18 or older with newly diagnosed AML at the Virginia Commonwealth University. Patients received frontline AZA-VEN or IC (7 + 3 cytarabine and anthracycline) to achieve their first CR before allogeneic peripheral blood stem-cell transplantation with posttransplant cyclophosphamide (PTCY), mycophenolate mofetil, and calcineurin inhibitor GVHD prophylaxis between January 1, 2023, and May 30, 2025. In the IC group, patients with FLT3 mutations received IC plus midostaurin. Treatment allocation to AZA-VEN or IC was physician-dependent.

Patients who received both AZA-VEN and IC to achieve their first CR were excluded. The Virginia Commonwealth University Institutional Review Board (Ethics Committee) approved this retrospective study. The study followed the 1964 Helsinki Declaration and its later amendments.

### Definitions

Patient, disease, and transplant characteristics were collected in an electronic database. The Hematopoietic Cell Transplantation-Specific Comorbidity Index (HCT-CI) was calculated as described by Sorror et al [14]. The AML diagnostic, genetic risk, response criteria, and relapse criteria followed the European Leukemia Net (ELN) 2022 guidelines [15]. Secondary AML was defined according to the ELN 2022 guidelines [15]. The Disease Risk Index (DRI) was defined according to Armand et al [16]. Myeloablative or reduced-intensity transplant conditioning intensity was defined as described by Bacigalupo et al [17]. Acute and chronic GVHD were defined according to consensus criteria [18–20].

### Minimal residual disease testing

Minimal residual disease (MRD) testing by multicolor flow cytometry (MFC) was performed within a month of transplantation and was sent to a reference laboratory at the University of Washington Medical Center Hematopathology Department in Seattle, USA, as previously described [21–23]. In brief, bone marrow aspirates were processed, stained with a three tube panel of titrated and optimized antibody-fluorochrome conjugates (tube 1: HLA-DR, CD15, CD33, CD19, CD117, CD13, CD38, CD34, CD71, and CD45; tube 2: HLA-DR, CD64, CD123, CD4, CD14, CD13, CD38, CD34, CD16, CD45; tube 3: CD56, CD7, CD5, CD33, CD39, CD34, CD45), and acquired on a three-laser, 12-color Becton Dickinson Lyric flow cytometer. Optimal stability is less than or equal to 72 h, with a goal of collecting 500,000 to 1,000,000 events. Data were analyzed using Woodlist v3.1.3 software using a “difference from normal” and “leukemia-associated immunophenotype” combination approach. During validation, assay sensitivity was conservatively estimated at 0.1% and, for some immunophenotypes, extended to 0.01%.

### Statistical analysis

This is a retrospective, single-center study. Patients with AML who received IC (cytarabine and anthracycline) were compared with those who received low-intensity therapy (AZA-VEN). Baseline patient, disease, and transplant characteristics of each cohort were summarized using descriptive statistics. Comparison of categorical variables used the Chi-square test or Fisher’s exact test. The Wilcoxon rank-sum test compared the continuous variables.

The primary outcomes of our study were OS and relapse-free survival (RFS). Secondary outcomes included GVHD-free, relapse-free survival (GRFS), and the cumulative incidences of non-relapse mortality (NRM), relapse, acute GVHD, and chronic GVHD. OS was defined as the time from stem cell infusion to death or last follow-up, with surviving patients censored at the time of the last follow-up. NRM was measured from stem cell infusion to death without prior relapse, with patients censored at last follow-up. RFS was measured from the stem cell infusion to the date of disease relapse/progression, death, or last follow-up. Patients alive without relapse at the last follow-up were censored. GRFS was determined from the stem cell infusion to the first occurrence of any of the following: grade III–IV acute GVHD, extensive chronic GVHD, relapse, or death. Patients who did not experience any of these events by the last follow-up were censored.

Survival probabilities (OS, RFS, and GRFS) were assessed by the Kaplan–Meier method. The differences between the groups were determined by the log-rank test. Associations between survival outcomes and potential prognostic factors, including induction therapy type, were evaluated using univariable and multivariable Cox proportional hazards regression models. All covariates were assessed for the proportional hazards assumption, and variables that violated this assumption were adjusted by stratification. The cumulative incidences of relapse, NRM, acute GVHD, and chronic GVHD were estimated using competing-risks methodology. The competing risk for NRM was relapse, and for relapse, it was death in CR. For GVHD endpoints, competing risks included death and relapse, without GVHD. Differences in cumulative incidence between cohorts were assessed using the Fine–Gray subdistribution hazards model. All analyses were performed utilizing the R version 4.4.2.

## Results

A summary of the baseline characteristics for the AZA-VEN and IC cohorts is listed in Table 1. The median age in the AZA-VEN (N = 14) and IC (N = 17) cohorts was comparable (67 years (61–69) vs. 63 years (54–66), P = 0.13). Nine patients with Fms-related tyrosine kinase (FLT3) internal tandem duplication (ITD)-mutated AML in the IC cohort received the FLT3 inhibitor midostaurin with anthracycline and cytarabine induction chemotherapy before transplant, followed by maintenance FLT3 inhibitors after transplant. No patient in the AZA-VEN group received a FLT3 inhibitor during induction chemotherapy. The median time from AML diagnosis to transplantation was similar in the two cohorts: 6.6 months (range, 4–14.1) in the AZA-VEN group and 6.1 months (range, 4–10.4) in the IC arm. The AZA-VEN group had greater adverse-risk AML and less intermediate-risk AML compared with the IC group (adverse risk: 93% vs. 41%; intermediate risk: 0 vs. 55%; overall P = 0.001). The AZA-VEN cohort had more patients with a high/very high DRI than the IC cohort (100% vs. 88%; overall P = 0.016). Otherwise, the cohorts were balanced with respect to Karnofsky performance status, HCT-CI, secondary AML, MRD status, myeloablative conditioning (MAC) or reduced-intensity conditioning (RIC), or donor type (Table 1).

**Table 1:** Patient Characteristics of the Intensive Chemotherapy Versus Azacitidine-Venetoclax Cohorts

Patient characteristics	Intensive chemotherapy (N = 17)	Azacitidine-venetoclax (N = 14)	P-value
Gender			0.55
Female	9 (53%)	5 (36%)	
Male	8 (47%)	9 (64%)	
Age at transplant, median (range) years	63 (54–66)	67 (61–69)	0.13
Karnofsky Performance status			0.7
90% or greater	4 (24%)	2 (14%)	
Less than 90%	13 (76%)	12 (86%)	
HCT-CI			0.7
Less than 3	14 (82%)	10 (71%)	
3 or more	3 (18%)	4 (29%)	
Acute myeloid leukemia type			0.15
*De novo*	16 (94%)	10 (71%)	
Secondary	1 (6%)	4 (29%)	
European Leukemia Network risk			0.001
Adverse	7 (41%)	13 (93%)	
Intermediate	9 (53%)	0 (0%)	
Favorable	1 (6%)	1 (7%)	
Disease Risk Index			0.02
Very high	0 (0%)	4 (29%)	
High	15 (88%)	10 (71%)	
Intermediate	2 (12%)	0 (0%)	
Minimal residual disease at transplant			0.4
Yes	4 (24%)	6 (43%)	
No	13 (76%)	8 (57%)	
Transplant conditioning intensity			0.2
Myeloablative	3 (18%)	0 (0%)	
Reduced intensity	14 (82%)	14 (100%)	
Donor type			0.4
Matched unrelated	9 (53%)	8 (57%)	
Mismatch unrelated	1 (6%)	3 (21.5%)	
Haploidentical	4 (23.5%)	3 (21.5%)	
Matched related	3 (17.5%)	0 (0%)	

HCT-CI: Hematopoietic Cell Transplantation Comorbidity Index.

**Figure 1:**
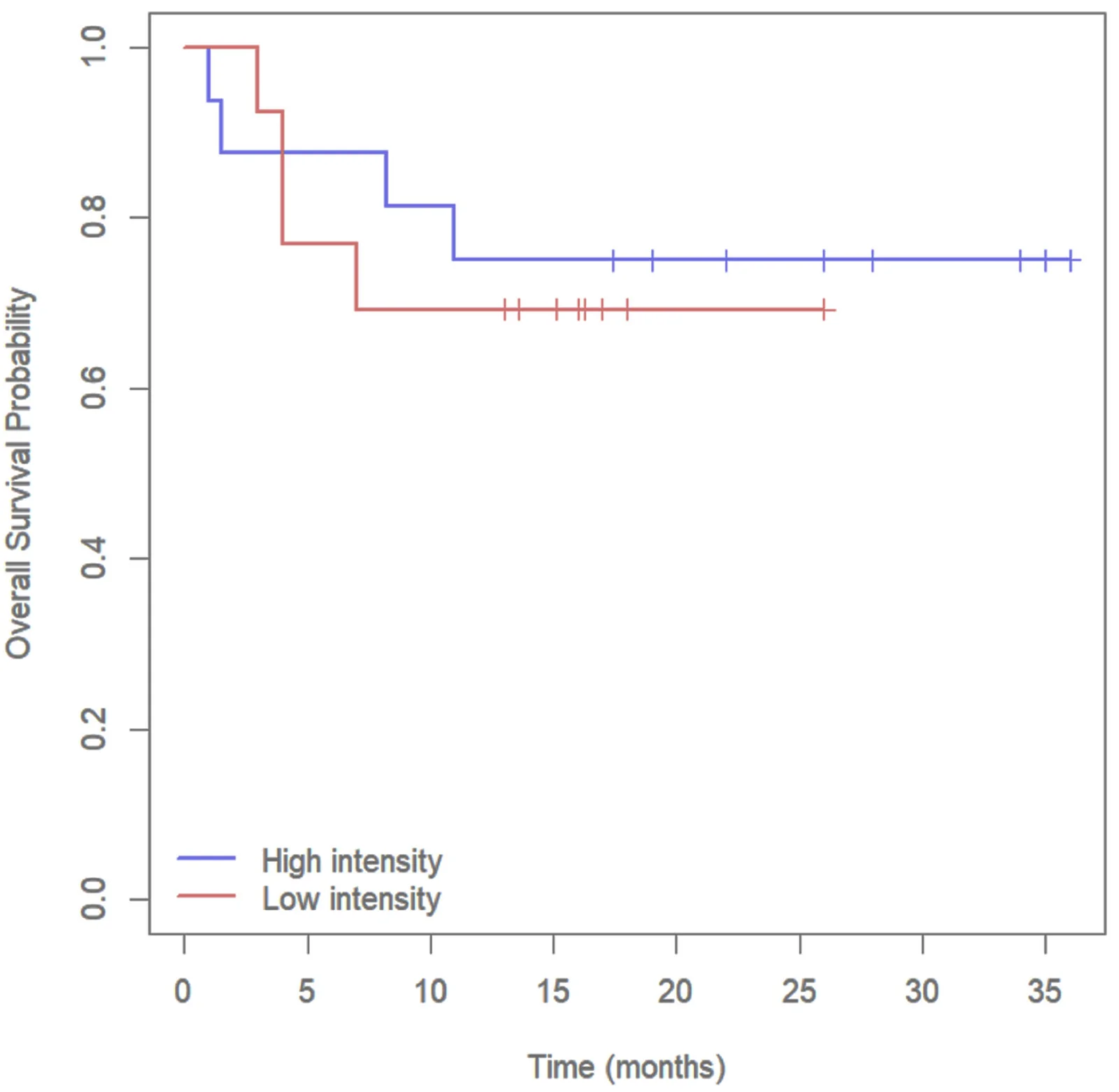
Kaplan–Meier estimates of overall survival in the intensive chemotherapy versus azacitidine-venetoclax cohorts (P = 0.7).

Median follow-up for the AZA-VEN and IC group was 14.35 months (range, 3–26) vs. 22 months (range, 1–36), respectively. During follow-up, four patients in each cohort died, with three patients dying from infection and one patient from relapse in each group. The CR rate with or without MRD negativity was similar in both cohorts (Table 2). The 1-year OS was comparable in AZA-VEN and IC cohorts (69.2%, 95% confidence interval (CI) (48.2–99.5) vs. 75%, 95% CI (56.5–99.5), P = 0.73) (Table 3, Fig. 1). Similarly, the 1-year RFS was similar for AZA-VEN and IC (57.1%, 95% CI (36.3–89.9) vs. 64.7%, 95% CI (45.5–91.9), P = 0.67) (Table 3).

**Table 2:** Outcomes of Allogeneic Stem-Cell Transplantation in the Intensive Chemotherapy Versus Azacitidine-Venetoclax Cohorts

Outcome after stem-cell transplant	Intensive chemotherapy	Azacitidine-venetoclax	P-value
Complete remission at day 100 posttransplant			0.7
CR/MRD negative	12 (71%)	12 (86%)	
CR/MRD positive	2 (12%)	1 (7%)	
Relapsed/deceased	3 (17%)	1 (7%)	
Acute graft-versus-host disease			0.6
Grade 0	4 (23.5%)	6 (43%)	
Grade I–II	9 (53%)	6 (43%)	
Grade III–IV	4 (23.5%)	2 (14%)	
Chronic graft-versus-host disease			0.2
None	7 (41%)	11 (79%)	
Limited	5 (29%)	1 (7%)	
Extensive	3 (18%)	1 (7%)	
Deceased	2 (12%)	1 (7%)	
Relapse			> 0.9
Yes	3 (18%)	3 (21%)	
No	14 (82%)	11 (79%)	
Deceased			> 0.9
Yes	4 (24%)	4 (29%)	
No	13 (76%)	10 (71%)	

CR: complete remission; MRD: minimal residual disease.

**Table 3:** Summary of 1-Year Estimates of Outcomes After Allogeneic Stem-Cell Transplantation in Intensive Chemotherapy Versus Azacitidine-Venetoclax Cohorts

Estimate at 1-year	Intensive chemotherapy	Azacitidine-venetoclax	P value
Overall survival (95% CI)	75% (56.5–99.5)	69.2% (48.2–99.5)	0.73
Relapse-free survival (95% CI)	64.7% (45.5–91.9)	57.1% (36.3–89.9)	0.67
Cumulative incidence of non-relapse mortality (%)	23.5	28.5	0.76
Cumulative incidence of relapse (%)	11.8	14.3	0.84
Cumulative incidence of acute GVHD II–IV (%)	23.5	35.7	0.48
Cumulative incidence of acute GVHD III–IV (%)	5.9	14.3	0.46
Cumulative incidence of chronic GVHD (%)	11.8	7.1	0.67
GRFS (95% CI)	64.7% (45.5–91.9)	57.1% (36.3–89.9)	0.67

CI: confidence interval; GVHD: graft-versus-host disease; GRFS: GVHD-free, relapse-free survival.

The 1-year cumulative incidence of relapse (14.3% vs. 11.8%, P = 0.84) and 1-year cumulative incidence of NRM (28.5% vs. 23.5%, P = 0.76) were not significantly different between the AZA-VEN or IC cohorts (Table 3). The cumulative incidences of grade 1–2 acute GVHD (21.4% vs. 17.6%, P = 0.80), grade 3–4 acute GVHD (14.3% vs. 5.9%, P = 0.46), or chronic GVHD (7.1% vs. 11.8%, P = 0.67) between the AZA-VEN and IC cohorts were not significantly different. The 1-year GRFS was not significantly different between the AZA-VEN and IC cohorts (57.1%, 95% CI (36.3–89.9) vs. 64.7% 95% CI (45.5–91.9), P = 0.67) (Table 3).

Univariable Cox proportional hazards models found no significant association between the individual covariates of treatment cohort (AZA-VEN vs. IC), age, MRD, adverse-risk genetics, acute GVHD, chronic GVHD, or HCT-CI and the OS, RFS, and GRFS for the two cohorts (Supplementary Materials 1–3, jh.elmerpub.com).

Fine–Gray subdistribution hazards models found no significant association between the individual covariates of treatment cohort (AZA-VEN vs. IC), MRD, adverse-risk genetics, chronic GVHD, or HCT-CI and the cumulative incidence of NRM for the two cohorts (Supplementary Material 4, jh.elmerpub.com). However, increasing age was significantly associated with a higher cumulative incidence of NRM (subdistribution hazard ratio (SHR) 1.20 per year, 95% CI (1.05–1.38), P = 0.01), but no significant difference was observed between cohorts AZA-VEN vs. IC (SHR 0.89, 95% CI (0.25–3.09), P = 0.88) (Supplementary Material 4, jh.elmerpub.com). Extensive chronic GVHD was associated with a greater cumulative incidence of NRM compared with no chronic GVHD (SHR 14.2, 95% CI (2.3–89.2), P = 0.005), and the AZA-VEN cohort was associated with a greater cumulative incidence of NRM compared with the IC cohort (SHR 5.44, 95% CI (1.31–22.6), P = 0.02) (Supplementary Material 4, jh.elmerpub.com). However, these estimates were accompanied by wide confidence intervals, likely reflecting the limited sample size, and therefore should be interpreted cautiously pending validation in larger studies.

Multivariable Cox proportional hazards analysis demonstrated no significant association between treatment cohort (AZA-VEN vs. IC) and OS (HR 2.27, 95% CI (0.19–26.82), P = 0.52), RFS (HR 3.30, 95% CI (0.61–18.01), P = 0.17), or GRFS (HR 4.45, 95% CI (0.85–23.2), P = 0.08). In addition, age, MRD status, adverse-risk genetics, acute GVHD, and HCT-CI were not significantly associated with OS in multivariable analysis (Supplementary Material 5(a), jh.elmerpub.com). Similarly, age, MRD status, GVHD, and HCT-CI were not significantly associated with RFS, NRM, or GRFS in multivariable analyses (Supplementary Material 5(b)–(d), jh.elmerpub.com).

To evaluate the potential impact of an imbalance in conditioning intensity between cohorts, we performed a sensitivity analysis by excluding the three patients in the IC cohort who received MAC, thereby restricting the analysis to patients receiving RIC only. Following exclusion of these patients, the overall study findings remain unchanged. One-year OS, RFS, cumulative incidence of relapse, NRM, and GVHD outcomes were comparable between the IC and AZA-VEN cohorts, consistent with the primary analysis (Supplementary Material 6(a), jh.elmerpub.com). In addition, treatment cohort effects remained non-significant in both Cox and Fine–Gray regression models, with HR estimates similar to those observed in the original analysis.

Baseline characteristics, transplant outcomes, and 1-year post-transplant outcomes were compared between FLT3-ITD–mutant and non-FLT3 AML patients within the IC cohort. Exploratory analyses did not demonstrate statistically significant differences in OS, RFS, NRM, or GVHD outcomes between the two subgroups (Supplementary Material 6(b)–(d), jh.elmerpub.com).

## Discussion

The outcomes (OS, RFS, and GRFS) of patients aged 18 or older with AML who received either AZA-VEN or IC (cytarabine and anthracycline) induction to achieve first CR before undergoing allogeneic peripheral blood stem-cell transplant were similar, irrespective of age, MRD status, or adverse-risk genetics. In addition, the 1-year cumulative incidences of relapse, NRM, and acute and chronic GVHD were similar in the AZA-VEN and IC cohorts. All patients had uniform GVHD prophylaxis with post-transplant cyclophosphamide, mycophenolate mofetil, and a calcineurin inhibitor. Of note, the AZA-VEN and IC cohorts did not differ significantly in Karnofsky performance status, HCT-CI, age, secondary AML, conditioning intensity, MRD status, donor type, or CR at day 100 post-transplant (Table 1).

In the PARADIGM study, AZA-VEN induction chemotherapy for newly diagnosed AML significantly improved composite CR and event-free survival despite a distribution of European Leukemia Net adverse-risk categories, including TP53 mutations, similar to that of the IC cohort [10]. Notably, the ELN 2022 genetic risk classification was developed in young AML patients receiving IC induction and was found not to be as prognostic in older patients receiving AZA-VEN induction [24]. However, by leveraging the graft-versus-leukemia effect, allogeneic HCT can overcome the poor prognosis associated with adverse-risk genetics in AML, such as secondary-type mutations *SF3B1, EZH2, STAG2, SRSF2, BCOR, U2AF1,* and *ZRSR2* [25, 26]. Our study also found similar outcomes in both cohorts after allogeneic HCT, despite the AZA-VEN group having a higher proportion of adverse-risk AML (93% vs. 41%, P = 0.001) and a more frequent high or very high DRI than the IC cohort (100% vs. 88%, P = 0.02). Similarly, patients in the PARADIGM study who reached transplant had comparable 1-year RFS, OS, and GVHD incidence, regardless of treatment cohort [10]. However, the PARADIGM study allowed patients in the IC arm to crossover to AZA-VEN and vice versa if they did not respond to induction. In contrast, our study excluded patients who had both IC and AZA-VEN to avoid confounding transplant outcomes.

In our study, MRD before allogeneic HCT did not affect OS or RFS in either treatment group (AZA-VEN or IC). However, the absence of MRD pre-transplant was associated with better outcomes after allogeneic HCT in some, but not all, studies [27–30]. For instance, in studies of newly diagnosed AML patients, MRD positivity before allogeneic HCT did not impact survival or relapse, particularly in patients older than 60 and those treated with azacitidine or decitabine and venetoclax [12, 28–30]. These findings suggest that the graft-versus-leukemia effect from allogeneic HCT may overcome MRD. Of note, the AZA-VEN cohort received RIC exclusively before transplant. Reduced-intensity conditioned allogeneic HCT relies on the graft-versus-leukemia effect to eliminate malignancy, in contrast to the direct tumor cell kill from myeloablative regimens [31].

This study is limited by its small sample size and retrospective design, which introduces inherent selection bias. Although follow-up was shorter in the AZA-VEN group (14.35 vs. 22 months for the IC cohort), it was adequate to detect most relapses, which usually occur in the first year after transplant [32]. Study strengths include consistent MRD testing at a reference laboratory in all patients, in contrast with prior studies [12, 13]. Also, patients received either AZA-VEN or IC without crossover or sequencing, unlike PARADIGM [10]. In addition, all patients received homogenous GVHD prophylaxis with post-transplant cyclophosphamide, mycophenolate mofetil, and a calcineurin inhibitor. Confounding variables, such as treatment cohort (AZA-VEN vs. IC), age, MRD status, adverse-risk genetics, acute GVHD, chronic GVHD, and HCT-CI, were analyzed and found not to significantly impact transplant outcomes (OS, RFS, and GRFS). These limitations suggest the need for prospective multicenter studies comparing AZA-VEN vs. IC induction in newly diagnosed AML prior to allogeneic HCT to confirm these findings. Despite these limitations and pending further validation in larger prospective studies, induction with AZA-VEN may be considered, especially in newly diagnosed AML patients with adverse-risk genetics, for whom the only possibility of a cure depends on getting to an allogeneic HCT.

In conclusion, AZA-VEN and IC induction for newly diagnosed AML yield comparable outcomes after allogeneic HCT, regardless of age, MRD status, or adverse-risk genetics, and should be explored in prospective studies.

## Supplementary Material

Suppl 1 Six Cox models used to analyze the impact of the individual covariates of treatment cohort (azacitidine-venetoclax versus intensive chemotherapy), age, minimal residual disease at transplant (MRD) before transplant, adverse risk genetics, acute GVHD, chronic GVHD, and Hematopoietic Cell Transplantation Comorbidity Index (HCT-CI) on the overall survival time distribution for the two cohorts (azacitidine-venetoclax versus intensive chemotherapy).

Suppl 2 Six Cox models used to analyze the impact of the individual covariates of treatment cohort (azacitidine-venetoclax versus intensive chemotherapy), age, minimal residual disease at transplant (MRD) before transplant, adverse risk genetics, acute GVHD, chronic GVHD, and Hematopoietic Cell Transplantation Comorbidity Index (HCT-CI) on the relapse-free survival time distribution for the two cohorts (azacitidine-venetoclax versus intensive chemotherapy).

Suppl 3 Six Cox models used to analyze the impact of the individual covariates of treatment cohort (azacitidine-venetoclax versus intensive chemotherapy), age, minimal residual disease before transplant (MRD), adverse risk genetics, acute graft-versus-host disease (GVHD), chronic GVHD, and Hematopoietic Cell Transplantation Comorbidity Index (HCT-CI) on the GVHD-free, relapse-free survival time.

Suppl 4 Six Fine–Gray subdistribution models used to analyze the impact of the individual covariates of treatment cohort (azacitidine-venetoclax versus intensive chemotherapy), age, minimal residual disease (MRD) before transplant, adverse risk genetics, acute GVHD, chronic GVHD, and Hematopoietic Cell Transplantation Comorbidity Index (HCT-CI) on the non-relapse mortality time distribution for the two cohorts (azacitidine-venetoclax versus intensive chemotherapy).

Suppl 5 (a) Multivariable Cox model used to analyze the impact of treatment cohort (azacitidine-venetoclax versus intensive chemotherapy), age, minimal residual disease before transplant (MRD), adverse risk genetics, acute graft-versus-host disease (GVHD), and Hematopoietic Cell Transplantation Comorbidity Index (HCT-CI) on the overall survival time distribution. (b) Multivariable Cox model used to analyze the impact of treatment cohort (azacitidine-venetoclax versus intensive chemotherapy), age, minimal residual disease before transplant (MRD), acute graft-versus-host disease (GVHD), chronic GVHD, and Hematopoietic Cell Transplantation Comorbidity Index (HCT-CI) on the relapse-free survival time distribution. (c) Multivariable Cox model used to analyze the impact of treatment cohort (azacitidine-venetoclax versus intensive chemotherapy), age, minimal residual disease before transplant (MRD), acute graft-versus-host disease (GVHD), and Hematopoietic Cell Transplantation Comorbidity Index (HCT-CI) on the non-relapse mortality time distribution. (d) Multivariable Cox model used to analyze the impact of treatment cohort (azacitidine-venetoclax versus intensive chemotherapy), age, minimal residual disease before transplant (MRD), acute graft-host-disease (GVHD), chronic GVHD, and Hematopoietic Cell Transplantation Comorbidity Index (HCT-CI) on the GVHD-relapse-free survival (GRFS) time distribution.

Suppl 6 (a) Summary of 1-year estimates of outcomes after allogeneic stem cell transplantation in intensive chemotherapy versus azacitidine-venetoclax cohorts after exclusion of myeloablative conditioning recipients. (b) Characteristics of FLT3-ITD–mutated versus non-FLT3 AML patients within the intensive chemotherapy cohort. (c) Outcomes of allogeneic stem cell transplantation FLT3-ITD–mutated versus non-FLT3 AML patients within the intensive chemotherapy cohort. (d) Summary of 1-year post-transplant outcomes in FLT3-ITD–mutated versus non-FLT3 AML patients within the intensive chemotherapy cohort.

## Data Availability

The authors declare that data supporting the findings of this study are available within the article.
