# Supplementary material for: The Impact of Induction With Azacitidine and Venetoclax Versus Intensive Chemotherapy on Outcomes After Allogeneic Hematopoietic Cell Transplantation for Newly Diagnosed Acute Myeloid Leukemia
Source: J Hematol. 2026 Aug 31;15(4):179–87. doi: 10.14740/jh2217 (PMC13568785; doi:10.14740/jh2217)
Supplement: Supplementary file 1 [file jh-15-04-179-s001.docx]

**Suppl 1.** Six Cox models used to analyze the impact of the individual covariates of treatment cohort (azacitidine-venetoclax versus intensive chemotherapy), age, minimal residual disease at transplant (MRD) before transplant, adverse risk genetics, acute GVHD, chronic GVHD, and Hematopoietic Cell Transplantation Comorbidity Index (HCT-CI) on the overall survival time distribution for the two cohorts (azacitidine-venetoclax versus intensive chemotherapy)

| **EFFECT** | **Degree of Freedom** | **Hazard ratio HR [95% CI]** | **P-value** |
| --- | --- | --- | --- |
| *COHORT* | 1 | 0.97 [0.26-4.00] | 0.97 |
| **Age** | 1 | 1.19 [0.10-1.43] | 0.05 |
| *COHORT* | 1 | 1.25 [0.31-5.06] | 0.75 |
| No MRD | 1 | 0.79 [0.19-3.34] | 0.75 |
| *COHORT* | 1 | 3.73 [0.35-39.6] | 0.27 |
| Intermediate vs. adverse risk genetics  Favorable vs. adverse risk genetics | 1  1 | 4.90 [0.39-61.8]  5.41 [0.48-60.4] | 0.22  0.17 |
| *COHORT*  Acute GVHD I-II vs. Acute GVHD 0  Acute GVHD III-IV vs. Acute GVHD 0 | 1  1  1 | 1.70 [0.38-7.63]  1.11 [0.18-6.80]  3.05 [0.45-20.9] | 0.49  0.91  0.26 |
| *COHORT*  Chronic GVHD limited vs. none  Chronic GVHD extensive vs. none | 1  1  1 | 6.24 [0.67-57.9]  2.69 [0.22-33.3]  15.0 [1.27-176] | 0.11  0.44  0.03 |
| *COHORT* | 1 | 1.22 [0.30-4.93] | 0.78 |
| HCT-CI ≥ 3 High risk | 1 | 1.87 [0.45-7.89] | 0.39 |
|  |  |  |  |
|  |  |  |  |
