# Supplementary material for: The Impact of Induction With Azacitidine and Venetoclax Versus Intensive Chemotherapy on Outcomes After Allogeneic Hematopoietic Cell Transplantation for Newly Diagnosed Acute Myeloid Leukemia
Source: J Hematol. 2026 Aug 31;15(4):179–87. doi: 10.14740/jh2217 (PMC13568785; doi:10.14740/jh2217)
Supplement: Supplementary file 2 [file jh-15-04-179-s002.docx]

**Suppl 2.** Six Cox models used to analyze the impact of the individual covariates of treatment cohort (azacitidine-venetoclax versus intensive chemotherapy), age, minimal residual disease at transplant (MRD) before transplant, adverse risk genetics, acute GVHD, chronic GVHD, and Hematopoietic Cell Transplantation Comorbidity Index (HCT-CI) on the relapse-free survival time distribution for the two cohorts (azacitidine-venetoclax versus intensive chemotherapy)

| **EFFECT** | **Degree of Freedom** | **Hazard ratio HR [95% CI]** | **P-value** |
| --- | --- | --- | --- |
| *COHORT* | 1 | 1.35 [0.45-3.99] | 0.59 |
| **Age** | 1 | 1.03 [0.96-1.11] | 0.46 |
| *COHORT* | 1 | 1.43 [0.48-4.21] | 0.52 |
| No MRD | 1 | 0.89 [0.29-2.67] | 0.83 |
| *COHORT* | 1 | 3.35 [0.60-18.8] | 0.17 |
| Intermediate vs. adverse risk genetics  Favorable vs. adverse risk genetics | 1  1 | 3.65 [0.60-22.2]  2.79 [0.29-26.8] | 0.16  0.37 |
| *COHORT*  Acute GVHD I-II vs. Acute GVHD 0  Acute GVHD III-IV vs. Acute GVHD 0 | 1  1  1 | 1.74 [0.54-5.64]  1.53 [0.43-5.48]  1.84 [0.36-9.45] | 0.36  0.51  0.46 |
| *COHORT*  Chronic GVHD limited vs. none  Chronic GVHD extensive vs. none | 1  1  1 | 2.16 [0.52-8.90]  0.82 [0.15-4.41]  1.97 [0.33-11.9] | 0.29  0.81  0.46 |
| *COHORT* | 1 | 1.46 [0.50-4.29] | 0.50 |
| HCT-CI ≥ 3 High risk | 1 | 0.88 [0.24-3.16] | 0.84 |
|  |  |  |  |
|  |  |  |  |
