# Supplementary material for: The Impact of Induction With Azacitidine and Venetoclax Versus Intensive Chemotherapy on Outcomes After Allogeneic Hematopoietic Cell Transplantation for Newly Diagnosed Acute Myeloid Leukemia
Source: J Hematol. 2026 Aug 31;15(4):179–87. doi: 10.14740/jh2217 (PMC13568785; doi:10.14740/jh2217)
Supplement: Supplementary file 3 [file jh-15-04-179-s003.docx]

**Suppl 3.** Six Cox models used to analyze the impact of the individual covariates of treatment cohort (azacitidine-venetoclax versus intensive chemotherapy), age, minimal residual disease before transplant (MRD), adverse risk genetics, acute graft-versus-host disease (GVHD), chronic GVHD, and Hematopoietic Cell Transplantation Comorbidity Index (HCT-CI) on the GVHD-free, relapse-free survival time.

| **EFFECT** | **Degree of Freedom** | **HR [95% CI]** | **P-value** |
| --- | --- | --- | --- |
| *COHORT* | 1 | 1.18 [0.42-3.32] | 0.75 |
| Age | 1 | 1.03 [0.97-1.10] | 0.33 |
| *COHORT* | 1 | 1.27 [0.45-3.58] | 0.65 |
| No MRD | 1 | 0.81 [0.30-2.23] | 0.69 |
| *COHORT* | 1 | 2.30 [0.58-9.07] | 0.23 |
| Intermediate vs. adverse risk genetics  Favorable vs. adverse risk genetics | 1  1 | 2.63 [0.73-9.52]  1.36 [0.15-12.0] | 0.14  0.78 |
| *COHORT*  Acute GVHD I-II vs. Acute GVHD 0  Acute GVHD III-IV vs. Acute GVHD 0 | 1  1  1 | 2.07 [0.65-6.64]  1.75 [0.50-6.11]  4.24 [1.02-17.7] | 0.22  0.38  0.05 |
| *COHORT*  Chronic GVHD limited vs. none  Chronic GVHD extensive vs. none | 1  1  1 | 1.69 [0.48-5.88]  0.60 [0.15-2.41]  1.49 [0.38-5.88] | 0.41  0.48  0.57 |
| *COHORT* | 1 | 1.36 [0.48-3.88] | 0.56 |
| HCT-CI ≥ 3 High risk | 1 | 0.69 [0.18-2.25] | 0.49 |
|  |  |  |  |
|  |  |  |  |
