# Supplementary material for: The Impact of Induction With Azacitidine and Venetoclax Versus Intensive Chemotherapy on Outcomes After Allogeneic Hematopoietic Cell Transplantation for Newly Diagnosed Acute Myeloid Leukemia
Source: J Hematol. 2026 Aug 31;15(4):179–87. doi: 10.14740/jh2217 (PMC13568785; doi:10.14740/jh2217)
Supplement: Supplementary file 4 [file jh-15-04-179-s004.docx]

**Suppl 4.** Six Fine-Gray subdistribution models used to analyze the impact of the individual covariates of treatment cohort (azacitidine-venetoclax versus intensive chemotherapy), age, minimal residual disease (MRD) before transplant, adverse risk genetics, acute GVHD, chronic GVHD, and Hematopoietic Cell Transplantation Comorbidity Index (HCT-CI) on the non-relapse mortality time distribution for the two cohorts (azacitidine-venetoclax versus intensive chemotherapy).

| **EFFECT** | **Degree of Freedom** | **SHR (subdistribution hazard ratio) [95% CI]** | **P-value** |
| --- | --- | --- | --- |
| *COHORT* | 1 | 0.89 [0.25-3.09] | 0.88 |
| Age | 1 | 1.20 [1.05-1.38] | 0.01 |
| *COHORT* | 1 | 1.21 [0.32-4.56] | 0.77 |
| No MRD | 1 | 0.87 [0.23-3.26] | 0.83 |
| *COHORT* | 1 | 3.48 [0.18-67.9] | 0.41 |
| Intermediate vs. adverse risk genetics  Favorable vs. adverse risk genetics | 1  1 | 4.40 [0.19-103]  5.71 [0.58-56.1] | 0.36  0.14 |
| *COHORT*  Acute GVHD I-II vs. Acute GVHD 0  Acute GVHD III-IV vs. Acute GVHD 0 | 1  1  1 | 1.66 [0.31-8.91]  1.19 [0.15-9.32]  3.34 [0.38-29.1] | 0.55  0.87  0.22 |
| *COHORT*  Chronic GVHD limited vs. none  Chronic GVHD extensive vs. none | 1  1  1 | 5.44 [1.31-22.6]  2.88 [0.29-28.8]  14.2 [2.25-89.2] | 0.02  0.37  0.005 |
| *COHORT* | 1 | 1.19 [0.32-4.36] | 0.80 |
| HCT-CI ≥ 3 High risk | 1 | 2.09 [0.57-7.68] | 0.27 |
|  |  |  |  |
|  |  |  |  |
