# Supplementary material for: The Impact of Induction With Azacitidine and Venetoclax Versus Intensive Chemotherapy on Outcomes After Allogeneic Hematopoietic Cell Transplantation for Newly Diagnosed Acute Myeloid Leukemia
Source: J Hematol. 2026 Aug 31;15(4):179–87. doi: 10.14740/jh2217 (PMC13568785; doi:10.14740/jh2217)
Supplement: Supplementary file 5 [file jh-15-04-179-s005.docx]

**Suppl 5. (a)** Multivariable Cox model used to analyze the impact of treatment cohort (azacitidine-venetoclax versus intensive chemotherapy), age, minimal residual disease before transplant (MRD), adverse risk genetics, acute graft-versus-host disease (GVHD), and Hematopoietic Cell Transplantation Comorbidity Index (HCT-CI) on the overall survival time distribution.

| **EFFECT** | **Degree of Freedom** | **Hazard ratio HR [95% CI]** | **P-value** |
| --- | --- | --- | --- |
| Cohort | 1 | 2.27 [0.19-26.8] | 0.52 |
| Age  No MRD  Intermediate vs. adverse risk genetics  Favorable vs. adverse risk genetics  Acute GVHD I-II vs. Acute GVHD 0  Acute GVHD III-IV vs. Acute GVHD 0  HCT-CI ≥ 3 High risk | 1  1  1  1  1  1  1 | 1.30 [0.95-1.79]  0.79 [0.10-6.40]  9.11 [0.35-240]  3.57 [0.19-66.5]  0.57 [0.05-6.29]  0.24 [0.01-6.95]  0.66 [0.11-4.12] | 0.10  0.83  0.19  0.39  0.65  0.40  0.65 |

**Suppl 5.** **(b)** Multivariable Cox model used to analyze the impact of treatment cohort (azacitidine-venetoclax versus intensive chemotherapy), age, minimal residual disease before transplant (MRD), acute graft-versus-host disease (GVHD), chronic GVHD, and Hematopoietic Cell Transplantation Comorbidity Index (HCT-CI) on the relapse-free survival time distribution.

| **EFFECT** | **Degree of Freedom** | **Hazard ratio HR [95% CI]** | **P-value** |
| --- | --- | --- | --- |
| Cohort | 1 | 3.31 [0.61-18.0] | 0.17 |
| Age  No MRD  Acute GVHD I-II vs. Acute GVHD 0  Acute GVHD III-IV vs. Acute GVHD 0  Chronic GVHD limited vs. none  Chronic GVHD extensive vs. none  HCT-CI ≥ 3 High risk | 1  1  1  1  1  1  1 | 0.99 [0.89-1.10]  0.75 [0.18-3.13]  1.77 [0.32-9.88]  4.07 [0.31-53.9]  0.88 [0.13-6.09]  2.48 [0.43-14.5]  1.02 [0.15-7.08] | 0.84  0.69  0.52  0.29  0.89  0.31  0.98 |

**Suppl 5. (c)** Multivariable Cox model used to analyze the impact of treatment cohort (azacitidine-venetoclax versus intensive chemotherapy), age, minimal residual disease before transplant (MRD), acute graft-versus-host disease (GVHD), and Hematopoietic Cell Transplantation Comorbidity Index (HCT-CI) on the non-relapse mortality time distribution.

| **EFFECT** | **Degree of Freedom** | **Hazard ratio HR [95% CI]** | **P-value** |
| --- | --- | --- | --- |
| Cohort | 1 | 0.92 [0.15-5.51] | 0.92 |
| Age  No MRD  Acute GVHD I-II vs. Acute GVHD 0  Acute GVHD III-IV vs. Acute GVHD 0  HCT-CI ≥ 3 High risk | 1  1  1  1  1 | 1.21 [0.97-1.51]  1.62 [0.27-9.67]  0.87 [0.10-7.83]  1.19 [0.07-21.0]  1.32 [0.28-6.17] | 0.09  0.60  0.90  0.90  0.73 |

**Suppl 5. (d)** Multivariable Cox model used to analyze the impact of treatment cohort (azacitidine-venetoclax versus intensive chemotherapy), age, minimal residual disease before transplant (MRD), acute graft-host-disease (GVHD), chronic GVHD, and Hematopoietic Cell Transplantation Comorbidity Index (HCT-CI) on the GVHD-relapse-free survival (GRFS) time distribution.

| **EFFECT** | **Degree of Freedom** | **Hazard ratio HR [95% CI]** | **P-value** |
| --- | --- | --- | --- |
| Cohort | 1 | 4.45 [0.85-23.2] | 0.08 |
| Age  No MRD  Acute GVHD I-II vs. Acute GVHD 0  Acute GVHD III-IV vs. Acute GVHD 0  Chronic GVHD limited vs. none  Chronic GVHD extensive vs. none  HCT-CI ≥ 3 High risk | 1  1  1  1  1  1  1 | 0.98 [0.89-1.08]  0.80 [0.21-2.99]  2.29 [0.40-13.0]  12.7 [1.07-151]  0.70 [0.15-3.25]  2.15 [0.50-9.29]  0.94 [0.14-6.14] | 0.65  0.74  0.35  0.04  0.65  0.30  0.95 |
