# Supplementary material for: The Impact of Induction With Azacitidine and Venetoclax Versus Intensive Chemotherapy on Outcomes After Allogeneic Hematopoietic Cell Transplantation for Newly Diagnosed Acute Myeloid Leukemia
Source: J Hematol. 2026 Aug 31;15(4):179–87. doi: 10.14740/jh2217 (PMC13568785; doi:10.14740/jh2217)
Supplement: Supplementary file 6 [file jh-15-04-179-s006.docx]

**Suppl 6. (a)** Summary of 1-year estimates of outcomes after allogeneic stem cell transplantation in intensive chemotherapy versus azacitidine-venetoclax cohorts after exclusion of myeloablative conditioning recipients.

| **Estimate at 1-year** | **Intensive Chemotherapy** | **Azacitidine-Venetoclax** | ***P* value** |
| --- | --- | --- | --- |
| Overall survival (95% CI) | 71.4% [51.3-99.5] | 69.2% [48.2-99.5] | 0.90 |
| Relapse free survival (95% CI) | 64.3% [43.5-95.0] | 57.1% [36.3-89.9] | 0.69 |
| Cumulative incidence of non-relapse mortality (%) | 28.5 | 28.5 | 1 |
| Cumulative incidence of relapse (%) | 7.14 | 14.3 | 0.56 |
| Cumulative incidence of acute GVHD II-IV (%) | 21.43 | 35.7 | 0.63 |
| Cumulative incidence of acute GVHD III-IV (%) | 7.14 | 14.3 | 0.56 |
| Cumulative incidence of chronic GVHD. (%) | 16.7 | 7.1 | 0.52 |
| GRFS (95% CI) | 64.3% [43.5-95.0] | 57.1% [36.3-89.9] | 0.69 |

**Suppl 6. (b)** Characteristics of FLT3-ITD–mutated versus mon-FLT3 AML patients within the intensive chemotherapy cohort

| Patient Characteristics | Non-FLT3 Mutation  N=8 | FLT3 Mutation  N=9 | *P*-value^1^ |
| --- | --- | --- | --- |
| Gender  Female  Male | 5 (62%)  3 (38%) | 4 (44%)  5 (56%) | 0.8 |
| Age at transplant  Median (range) years | 66 (63-67) | 60 (49-64) | 0.06 |
| Karnofsky Performance status  90% or greater  Less than 90% | 1 (13%)  7 (87%) | 3 (33%)  6 (67%) | 0.6 |
| HCT-CI  Less than 3  3 or more | 7 (88%)  1 (12%) | 7 (78%)  2 (22%) | >0.9 |
| Acute myeloid leukemia type  De novo  Secondary | 7 (88%)  1 (12%) | 9 (100%)  0 (0%) | 0.5 |
| European Leukemia Network risk  Adverse  Intermediate  Favorable | 5 (63%)  2 (25%)  1 (12%) | 2 (22%)  7 (78%)  0 (0%) | 0.081 |
| Disease Risk Index  Very high  High  Intermediate | 0 (0%)  7 (88%)  1 (12%) | 0 (0%)  7 (89%)  1 (11%) | >0.9 |
| Minimal Residual Disease at transplant  Yes  No | 2 (25%)  6 (75%) | 2 (22%)  7 (78%) | >0.9 |
| Transplant Conditioning Intensity  Myeloablative  Reduced intensity | 0 (0%)  8 (100%) | 3 (33%)  6 (67%) | 0.2 |
| Donor Type  Matched unrelated  Mismatch unrelated  Haploidentical  Matched related | 5 (63%)  1 (13%)  1 (13%)  1 (13%) | 4 (44%)  0 (0%)  3 (33%)  2 (22%) | 0.7 |

**Suppl 6. (c)** Outcomes of allogeneic stem cell transplantation FLT3-ITD–mutated versus non-FLT3 AML patients within the intensive chemotherapy cohort

| **Outcome after stem-cell transplant** | **Non-FLT3 Mutation**  **N=8** | **FLT3 Mutation**  **N=9** | **P-value** |
| --- | --- | --- | --- |
| Complete remission at day 100 posttransplant  CR/MRD negative  CR/ MRD positive  Relapsed/deceased | 4 (50%)  2 (25%)  2 (25%) | 8(89%)  0 (0%)  0 (11%) | 0.2 |
| Acute Graft-versus-host disease  Grade 0  Grade I-II  Grade III-IV | 1 (12%)  5 (63%)  2 (25%) | 3 (34%)  4 (44%)  2 (22%) | 0.8 |
| Chronic Graft-versus-host disease  None  Limited  Extensive  Deceased | 3 (37.5%)  3 (37.5%)  0 (0%)  2 (25%) | 4 (45%)  2 (22%)  3 (33%)  0 (0%) | 0.2 |
| Relapse  Yes  No | 1 (12%)  7 (88%) | 2 (22%)  7 (78%) | >0.9 |

**Suppl 6. (d)** Summary of 1-year post-transplant outcomes in FLT3-ITD–mutated versus non-FLT3 AML patients within the intensive chemotherapy cohort

| **Estimate at 1-year** | **Non-FLT3 mutation** | **FLT3 mutation** | ***P* value** |
| --- | --- | --- | --- |
| Overall survival (95% CI) | 75.0% [50.3-1.00] | 75.0% [50.3-1.00] | 1 |
| Relapse free survival (95% CI) | 75.0% [50.3-1.00] | 55.6% [31.0-99.7] | 0.38 |
| Cumulative incidence of non-relapse mortality (%) | 25.0 | 22.2 | 0.90 |
| Cumulative incidence of relapse (%) | 0.00 | 22.2 | 0.13 |
| Cumulative incidence of acute GVHD II-IV (%) | 12.5 | 33.3 | 0.62 |
| Cumulative incidence of acute GVHD III-IV (%) | 0.00 | 11.1 | 0.33 |
| Cumulative incidence of chronic GVHD. (%) | 0.00 | 22.2 | 0.14 |
| GRFS (95% CI) | 75.0% [50.3-1.00] | 55.6% [31.0-99.7] | 0.38 |
